# Co-analysis of methylation platforms for signatures of biological aging in the domestic dog reveals previously unexplored confounding factors

**DOI:** 10.18632/aging.206012

**Published:** 2024-07-09

**Authors:** Aitor Serres Armero, Reuben M. Buckley, Lajoyce Mboning, Gabriella J. Spatola, Steve Horvath, Matteo Pellegrini, Elaine A. Ostrander

**Affiliations:** 1 Cancer Genetics and Comparative Genomics Branch, National Human Genome Research Institute, National Institutes of Health, Bethesda, MD 20892, USA; 2 Department of Chemistry and Biochemistry, University of California Los Angeles, Los Angeles, CA 90095, USA; 3 Department of Human Genetics, David Geffen School of Medicine, University of California, Los Angeles, CA 90095, USA; 4 Altos Labs Inc, Cambridge, United Kingdom; 5 Department of Molecular, Cell and Developmental Biology, University of Los Angeles, Los Angeles, CA 90095, USA

**Keywords:** biological age, methylation, dog, lifespan, penalized regression

## Abstract

Chronological age reveals the number of years an individual has lived since birth. By contrast, biological age varies between individuals of the same chronological age at a rate reflective of physiological decline. Differing rates of physiological decline are related to longevity and result from genetics, environment, behavior, and disease. The creation of methylation biological age predictors is a long-standing challenge in aging research due to the lack of individual *pre-mortem* longevity data. The consistent differences in longevity between domestic dog breeds enable the construction of biological age estimators which can, in turn, be contrasted with methylation measurements to elucidate mechanisms of biological aging. We draw on three flagship methylation studies using distinct measurement platforms and tissues to assess the feasibility of creating biological age methylation clocks in the dog. We expand epigenetic clock building strategies to accommodate phylogenetic relationships between individuals, thus controlling for the use of breed standard metrics. We observe that biological age methylation clocks are affected by population stratification and require heavy parameterization to achieve effective predictions. Finally, we observe that methylation-related markers reflecting biological age signals are rare and do not colocalize between datasets.

## INTRODUCTION

Understanding the epigenomics of organismal aging is among the greater challenges in mammalian biology. Intracellular effects such as telomere shortening, double-strand breaks and the slowing of metabolism, as well as changes in cell fractions, display an age-dependent interplay between DNA methylation and histone modifications which result in changes in chromatin structure and gene expression [1]. These processes possess the ability to track important stages in the lifespan of an organism, a property commonly referred to as epigenetic age or epigenetic clock. Out of the many markers that can be used to create epigenetic clocks, DNA methylation has had the most widespread applicability across vertebrates [2], with recent publications achieving highly accurate methylation-based age predictions across numerous mammalian species [3–7].

**Figure 1:**
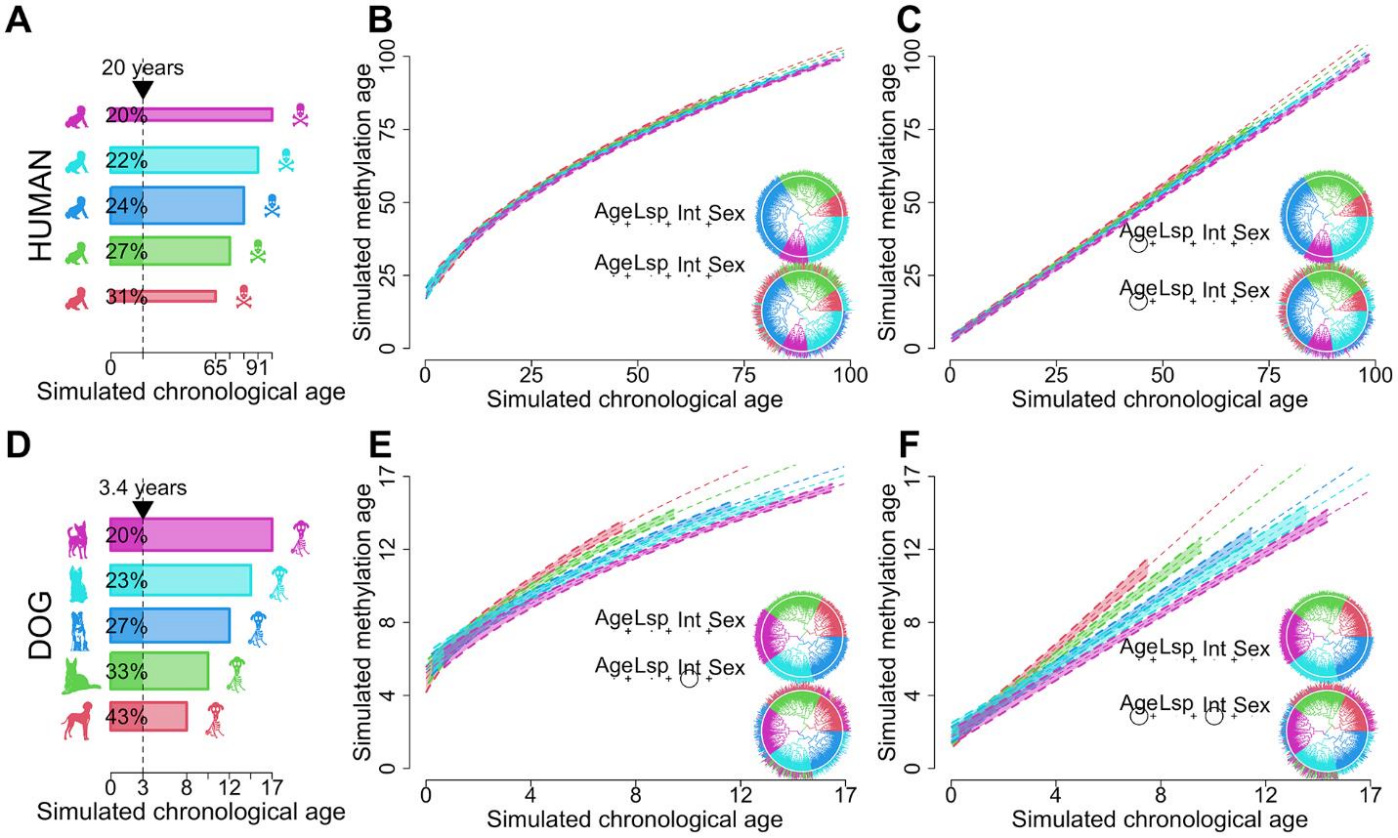
**Schematic showcasing the potential of dogs as models for biological aging.** Humans (**A**–**C**) are compared to dogs (**D**–**F**). Panels (**A**, **D**) represent idealized distributions of longevity. Human longevity (**A**) is centered around a particular value while dogs with infrequent extremes as depicted by the width of the bars. Dog longevity (**D**) is more evenly distributed across different breeds. The different colors represent longevity bins. The vertical dotted line in (**A**, **D**) shows the variation in biological ages for a fixed chronological age. The longevity distributions in (**A**, **D**) were used to generate the biological age regression models in (**B**, **C**, **E**, **F**). Ages were sampled uniformly from within the bounds of each longevity category. Regression models contain a dependent term raised to the second power and simulated values for age (Age), lifespan (Lsp), sex (Sex) and the quotient between age and longevity representing biological age (Int) as independent variables. The same coefficients, sample sizes and error distributions were used for humans and dogs, and phylogenies were randomly generated. Panels (**B**, **E**) represent the effects of non-linearity while (**C**, **F**) depict the linearized regression model. The p-values of each regression term are shown as circles in the inscribed equations with size proportional to -log magnitude, joined by plus signs to evoke linear regression. In panels (**B**, **C**, **E**, **F**) the top equations showcase instances where longevity is completely correlated with tree topology, as depicted by the matching color of the tree leaves and edges. In the bottom equations longevity has a smaller phylogenetic signal while still retaining some phylogenetic structure. The 99% confidence intervals and trend lines in the plots are produced by this second model.

Despite the evident success of DNA methylation at empirical predictions of age, disentangling the molecular mechanisms that underlie the interplay between methylation and aging at the epigenomic level remains a major challenge. A crucial step towards this goal is to determine whether the methylome mirrors aging chronologically, biologically, or both [8, 9]. “Chronological” aging defines the process by which all individuals age at the same speed. Therefore, chronological age refers to how much time has passed since birth and all individuals born on the same date will share the same chronological age, regardless of their expected longevity. “Biological” aging, also known as “functional” aging, defines the process by which individuals age at different speeds depending on their physiology, genetics, environmental exposures, behavioral choices, and other morbidity-related factors. While there is no consensus metric for measuring biological age, most definitions agree that the term encompasses factors that ultimately modulate the lifespan of an individual [10] (hereby also referred to as longevity or time of death). Consequently, in this study, biological age is defined as the quotient between chronological age and expected longevity (Figure 1). *Pre-mortem* estimates of longevity are unavailable for most organisms and therefore methylation-based insights into biological age are of the utmost interest to studies of longevity and quality of life [11]. Additionally, biological age permits the alignment of aging stages across species [12–14]. However, the power to differentiate between biological and chronological age decreases if organisms with similar lifespans are compared, as is the case with most intra-species methylation experiments (Figure 1). Additionally, intra-species biological age comparisons can be confounded by environmental factors and disease propensities, which are not necessarily the desired driving factors of a biological age epigenetic clock in healthy individuals.

Dog breeds are a valuable system for studying methylation in the context of longevity. Not only are meticulous birth records kept for all registered dogs as well as many mixed breeds, but purebred dogs display reproducible and significant lifespan differences which are associated with breed [15]. As such, by knowing the chronological age and breed of a collection of dog samples from multiple breeds, it is possible to estimate the order in which they will pass away with relative accuracy, a much less feasible endeavor in other domestic species such as cats, horses, or cattle [16–18]. Additionally, the difference in breed-based lifespan estimates is not explained by late onset disease propensities and established behaviors, as evidenced by the fact that short- and long-lived breeds have differentiated aging stages, such as time of puberty [19]. Finally, the largely monophyletic nature of most modern dog breeds [20, 21] makes it possible to perform population stratification correction of purebred dog datasets by utilizing previously established phylogenetic relationships, even if no genomic data pertaining to the specific samples is available. Recent studies [22, 23] highlight the interdependence of methylation measurements and genomic sequence composition at an interspecies level, underlining the relevance of phylogenetic correction in methylation studies. Previous analyses of DNA methylation in dogs and wolves have found success in constructing chronological age clocks, but report relatively small biological age effects [3, 24], a surprising outcome considering that species with much less variation in lifespan, such as humans, display biological age signals [25]. However, constructing chronological age clocks was the primary objective of most previous studies of dog aging, and the application of conventional statistical methods to a fixed subset of sites associated with chronological age may have hindered the construction of efficient biological age clocks in favor of chronological age effects.

**Figure 2:**
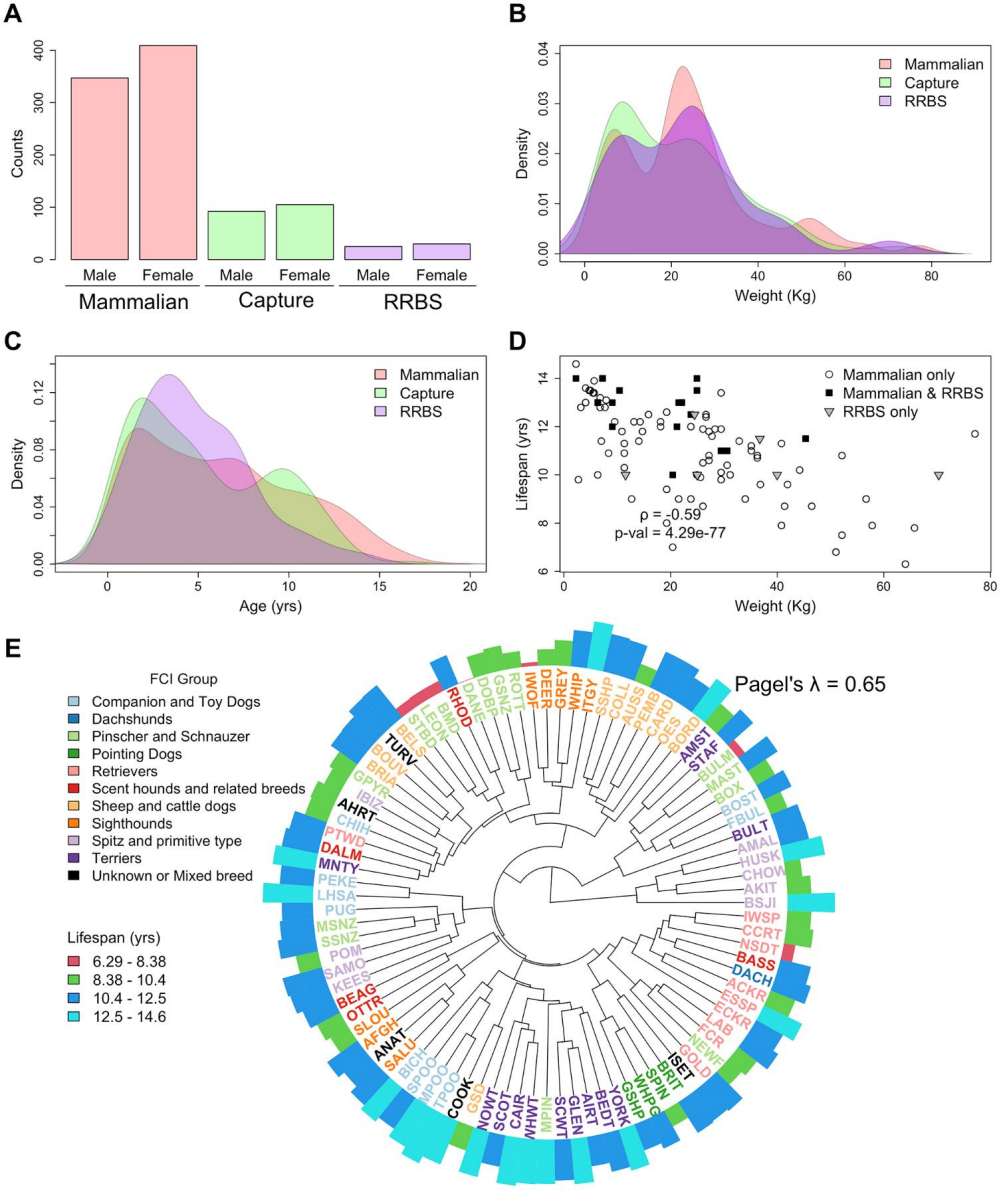
**Summary of the relevant phenotypes across the RRBS, mammalian methylation array and capture sequencing datasets.** (**A**–**C**) Distribution of sex, weight, and age in the three datasets. (**D**) Correlation between lifespan and weight in all breeds represented in the three studies. Capture sequencing contained a subset of the breeds represented in the mammalian methylation array. (**E**) Canonical phylogeny of the breeds represented in all three studies [20]. The length of the outer ring bars is proportional to lifespan and colored according to a binning of the lifespan distribution in equal length intervals. The tree tips are colored according to the Fédération Cynologique Internationale [35] (FCI) group and named after the convention in Parker et al. [20].

In this study we explore the potential of the three largest, publicly available DNA methylation datasets in dogs to identify signals of biological age. The featured studies include reduced representation bisulfite sequencing in dog peripheral blood [24] (henceforth referred to as RRBS) [number of markers: 244,333; 55 samples], an application of the well-validated mammalian methylation array [3] based on highly conserved regions across mammals tested using dog peripheral blood [number of markers: 30,930; 756 samples], and a capture sequencing experiment based on conserved and dog-specific loci tested using dog saliva [26] [number of markers: 4,341; 197 samples]. We also analyzed the data substituting lifespan for breed standard weight to estimate biological age as a way to ensure comparability across datasets, acknowledging that weight and lifespan are highly anticorrelated in dog breeds [15] (Figure 2). By virtue of applying a novel, generalized-error extension of the most commonly used penalized regression algorithms [3, 27], the clocks presented here are inherently corrected for phylogeny and unrestricted to any subset of sites (Methods, Supplementary Text 1 and Supplementary Figures 9, 10). Working on a phylogenetically independent framework enables the comparison of different methylation platforms and creates the potential to construct biological age clocks which are not confounded by neutral sequence variation. Additionally, we correct the methylation datasets for more traditional confounders such as sex and non-linearity. Finally, we evaluate biological age in a site-by-site manner correcting for all the above-mentioned confounders. The orthogonality of platforms and samples as well as the presence of two different tissues enables an assessment of biological age determination potential in dogs and encourages the evaluation of different methylation platforms.

## RESULTS

### A framework to detect biological age in the dog

We define biological age to be the quotient between the age of a sample and its inferred lifespan based on breed averages (Figure 1). To account for datasets containing mixed-breed samples, where no average breed lifespan could be inferred, we additionally defined biological age to be the product between the sample age and its individual weight, as lifespan and weight are generally anticorrelated in the dog [15] (Figure 2). These definitions can be statistically formalized as an interaction term between chronological age and lifespan or weight (hereby also referred to as moderators or moderator variables), and have some key differences with the model containing only the moderator variables as a main effect [8, 28] (Figure 1). The interaction term implies a changing rate of methylation as it correlates with age and longevity or weight (Figure 1). Conversely, the presence of significant moderators likely indicates that the methylation values at a site are associated with lifespan or weight, but independent of age. Furthermore, the known non-linear relationships between methylation and age may also mask interaction effects and produce inaccurate summary statistics (Figure 1). At a single site level, nonlinearities are overcome by using power transformations [29–31] or nonparametric approaches such as BayesAge [32] (Methods), while at a whole methylome level the epigenetic pacemaker theory developed by Snir et al. [33] ensures that a non-linearity trend is estimated globally.

### Dog breed standard lifespans and weights display high phylogenetic signals

Our analysis makes use of three previously published dog methylation datasets, each with differing breed compositions and sample size, featuring three distinct methylation platforms and two tissue types. The three studies have a well-balanced composition of males and females and a similar distribution of ages and weights (inter quantile ranges 2.49 ± 0.50 – 8.41 ± 1.26 years (yrs) and 10.27 ± 1.14 - 29.47 ± 0.10 kg, respectively, and Figure 2). Importantly, the capture sequencing dataset was composed of both purebred and mixed-breed dogs, and therefore lifespan values could not be assigned to all dogs. However, empirical weight measurements were available for all mixed-breed dogs in the dataset. Only 11 mixed-breed dogs of the185 had not reached maturity at the time indicated by the study metadata. The weights of all purebred dogs, including those that had not reached adulthood, were replaced with their respective breed standard adult weights [3]. We replicate the previously observed anticorrelation [15] between breed weights and lifespans for the breeds present in all datasets (ρ=-0.59 and F-test p-value=4.29e-77), and note that the relationship holds for each dataset individually (Figure 2). The use of standard breed weights and lifespans can obscure environmental effects such as overfeeding or under-exercising, which affect the weight and longevity of individual dogs. Both lifespan and weight displayed highly significant phylogenetic signals compared to the canonical dog breed phylogeny (Pagel’s [34] λ = 0.65 and 1 respectively). This highlights the difficulty encountered in decoupling not only lifespan from weight but also the effects of population stratification from both traits.

**Figure 3:**
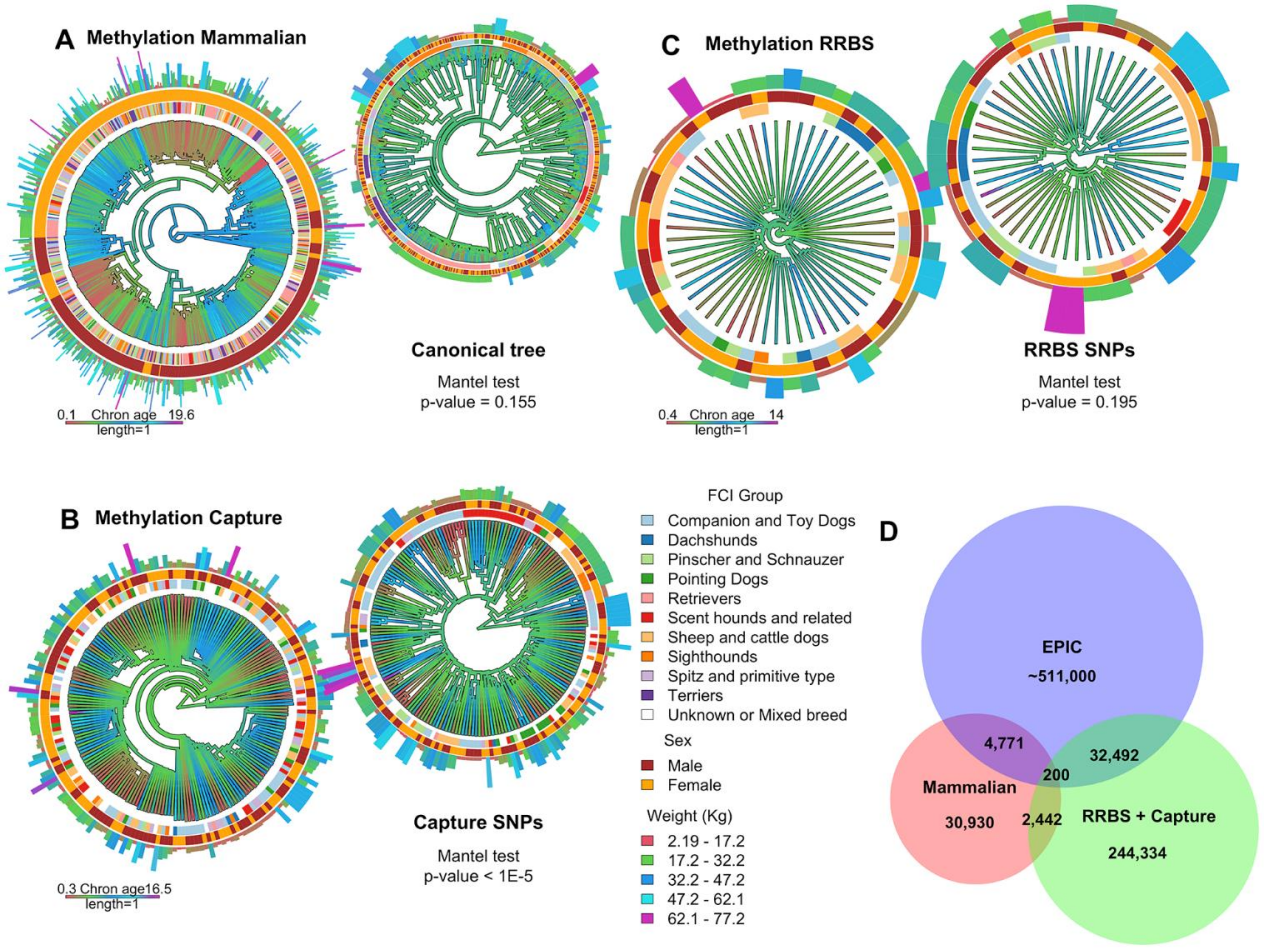
**Tree representation of the capture sequencing, mammalian methylation array and RRBS methylation datasets.** (**A**–**C**) The edges of each tree are colored with a gradient according to the age of each sample. The smaller trees at the top right corner of each panel correspond to genetic distance where age is also represented as a color gradient. The color of the innermost ring of the bigger and smaller trees corresponds to FCI clade, the middle ring corresponds to sex, and the length and color of the outermost bars to weight. (**D**) Venn diagram depicting the number of intersecting loci between the three studies. This also includes probes from the human EPIC commercial array [37], a methylation array platform commonly used in human studies, which align to the dog genome.

We also sought to determine how many sites were shared across the different datasets. However, the sparsity of array sites and capture experiments in addition to the stochastic dropout of lower depth sequencing could lead to an underestimation of shared sites. To make the comparison between arrays and sequencing data more less strict, we extended the genomic range of every site in every dataset 50 bps upstream and downstream and merged any overlapping loci. We postulate that neighboring methylation sites generally belong to larger scale elements, such as CpG islands or SINEs, and therefore have dependent methylation values. Although even a single bp overlap between datasets was considered a match, we observed very little overlap between platforms, suggesting that fully concordant cross-platform results are not necessarily expected (Table 1 and Figure 3).

**Table 1:** Table of genetic elements intersected by methylation sites in the mammalian methylation array and RRBS, on which the capture sequencing experiment is based.

	**Mammalian**	**RRBS and capture**	**Avg %methylation^a^**	**CanFam3.1(Promoters)**
**CpG Islands(Promoters)^b^**	1,680(964)	33,938(9,418)	55.01	**48,192(14,795)**
**First exons**	578	13,223	34.77	**40,266**
**Exons, not first**	10,339	26,657	77.27	**539,872**
**3UTR**	636	3,142	77.75	**31,720**
**5UTR**	1,154	5,991	25.69	**41,499**
**Introns**	8,998	122,255	73.66	**208,652**
**Intergenic**	7,250	108,123	71.05	**28,458**
**Union all^c^**	20,742	236,090	-	-

^a^Evenly weighted sum of the mean methylation measurement of any element in the mammalian and RRBS/capture datasets.

^b^Parentheses refer to how many of the CpG islands intersect promoters.

^c^Contains the union of all unique elements in a dataset, considering that some elements may belong to more than one category.

All three studies were able to independently derive chronological age clocks and determine sex from methylation data. However, consistent with the absence of shared markers among datasets, there were notable differences in major axes of variation. The mammalian methylation array was still able to predict sex even after removing any sites located in sex chromosomes but performed poorly at breed determination based on a methylation distance matrix (Mantel test p-value 0.155) (Figure 3). Conversely, capture sequencing offered a much more accurate recapitulation of phylogeny (Mantel test p-value <1e-5), which could be validated using SNPs extracted from the same dataset. Average RRBS methylation distances did not recapitulate phylogeny (Mantel test p-value 0.195), but genetic variation could still be extracted from the dataset using non-methylated bases (Methods).

### Sparse, biological age epigenetic clocks are confounded by phylogeny

**Figure 4:**
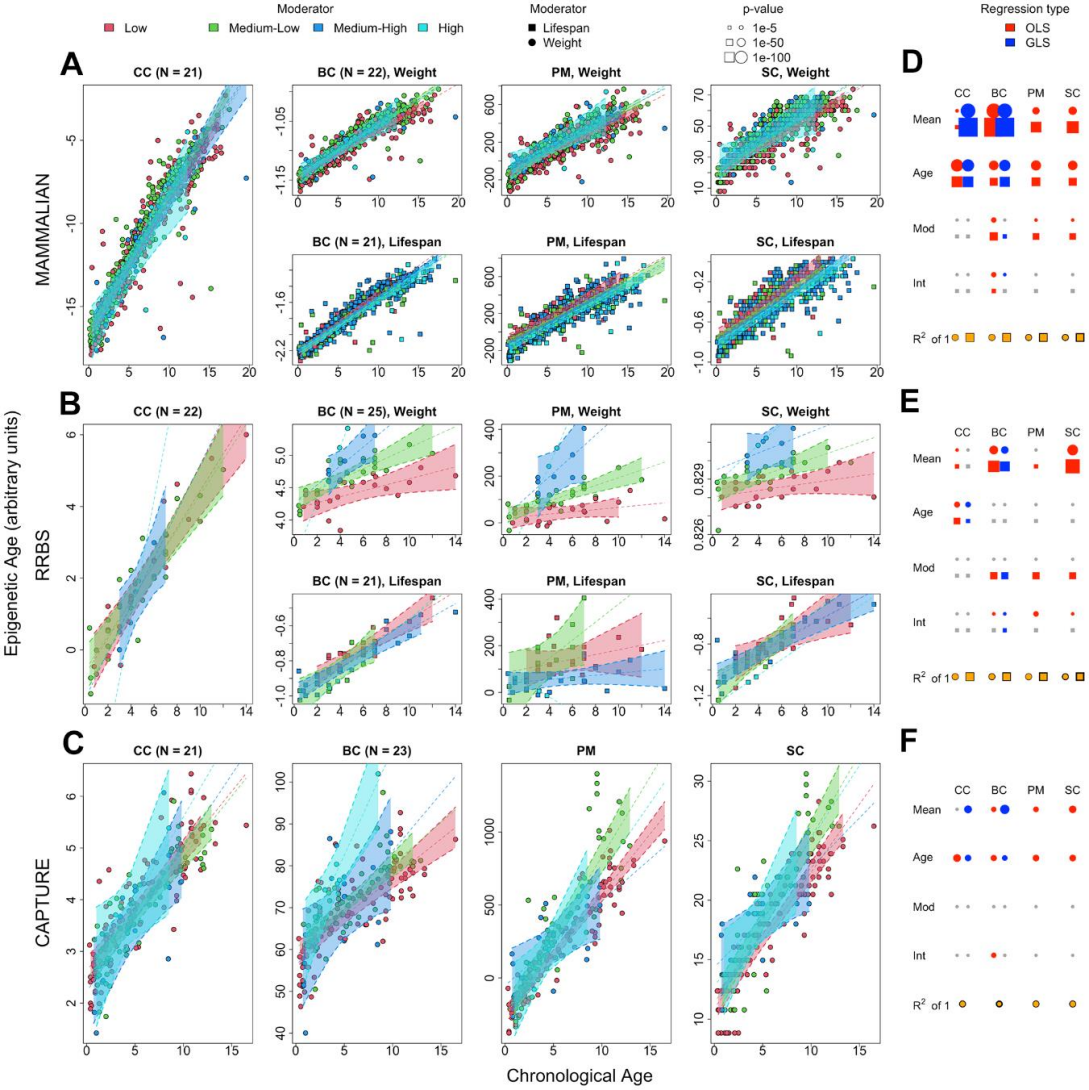
**Statistical analyses of chronological and biological age sparse epigenetic clocks using phylogenetic penalized regression.** Rows correspond to the mammalian methylation array, RRBS, and capture sequencing datasets, respectively. (**A**–**C**) The first, second, third and fourth panels in each row represent the different epigenetic clocks. CC: penalized generalized least squares regression trained on chronological age. BC: penalized generalized least squares regression trained on biological age (product of age and weight), PM: epigenetic pacemaker trained on biological age data, SC: BayesAge algorithm trained on biological age data. The trend lines and 99% confidence intervals are derived from the penalized, phylogenetic least squares prediction model. Any split panels depict the use of weight or lifespan as a moderator as described in the panel and legend. (**D**–**F**) The rightmost plots of each row depict the significance of each regressor in the corresponding dataset, with circle radii proportional to -log p-value (blue: phylogeny corrected least squares, red: ordinary least squares, gray: non-significant), the yellow-colored fraction of the area of the bottom circles and squares depicts the regression R^2^ values derived from the penalized, phylogenetic least squares prediction model.

We next created biological age clocks moderated by lifespan and weight and corrected for phylogenetic relationships between samples. In order to enhance comparability across datasets we limited the number of markers that make up any clock to ≤ 25, as this value lies in the interval between the number of markers corresponding to the minimum cross validating penalty value and the penalty value one standard error above it (Figure 4 and Supplementary Figure 1, and Methods). We posit that marker sparsity in biological age clocks can prevent overfitting and promote the inclusion of sites with large biological age effects (Supplementary Figure 1). Additionally, the ability to create sparse, cross-validating chronological age clocks in all datasets (Figure 4) motivated the assessment of such clocks for biological age. Of note, all biological and chronological age epigenetic clocks passed ten-fold cross-validation both under ordinary and generalized least squares error models except for RRBS moderated by weight (Figure 4C, 4D and Supplementary Figure 1). Finally, we applied the epigenetic pacemaker and BayesAge clocks [32] to the methylation data and regressed the output methylation state curves against biological age.

Using phylogenetically corrected error models while enforcing sparsity offered key insights into the interpretation of biological age across the three datasets (Figure 4). A qualitative comparison of ordinary least squares sparse models to their phylogenetically corrected analogs suggests a stark suppression regarding significance of the interaction terms in both the mammalian methylation array and capture sequencing datasets (Figure 4B, 4D, 4F, second column). In the case of RRBS, phylogenetic errors hampered cross validation (Supplementary Figure 1). This demonstrates that we cannot construct reliable, sparse biological age clocks that are uncounfounded by phylogeny using weight or lifespan as a moderator. Non-sparse biological age clocks generated using minimum cross-validating penalty values (Methods and Supplementary Figures 1, 2) included a variable number of markers and therefore were much less comparable. Notably, the mammalian methylation array required up to 264 markers to achieve its optimal biological age prediction power, and visual examination of those markers revealed poor correlation between individual sites and biological age (Supplementary Figure 3). In contrast, the capture sequencing epigenetic clock required 45 markers and retained a significant yet attenuated biological age signal even under the phylogenetically corrected error model.

Biological age clocks constructed using breed standard weight outperformed those constructed using lifespan across all datasets, even under sparse conditions and after correcting for phylogeny (Figure 4). Similarly, ordinary least squares clocks qualitatively outperformed those constructed using phylogenetic least squares, suggesting that the relationship between breed standard weights and lifespans and aging rates is often concordant with phylogenetic tree relationships. The epigenetic pacemaker and BayesAge models revealed small but significant biological age effects in almost all datasets. However, these effects could not be decoupled from phylogeny and a decorrelating transformation of the methylation matrices akin to that used in our generalized, penalized regression model yielded no valid results. As such, the epigenetic pacemaker and BayesAge predictions were deemed more comparable to penalized, ordinary least squares regression models than those that were phylogenetically corrected.

### Biological age methylation markers are scarce and display low effect sizes

**Figure 5:**
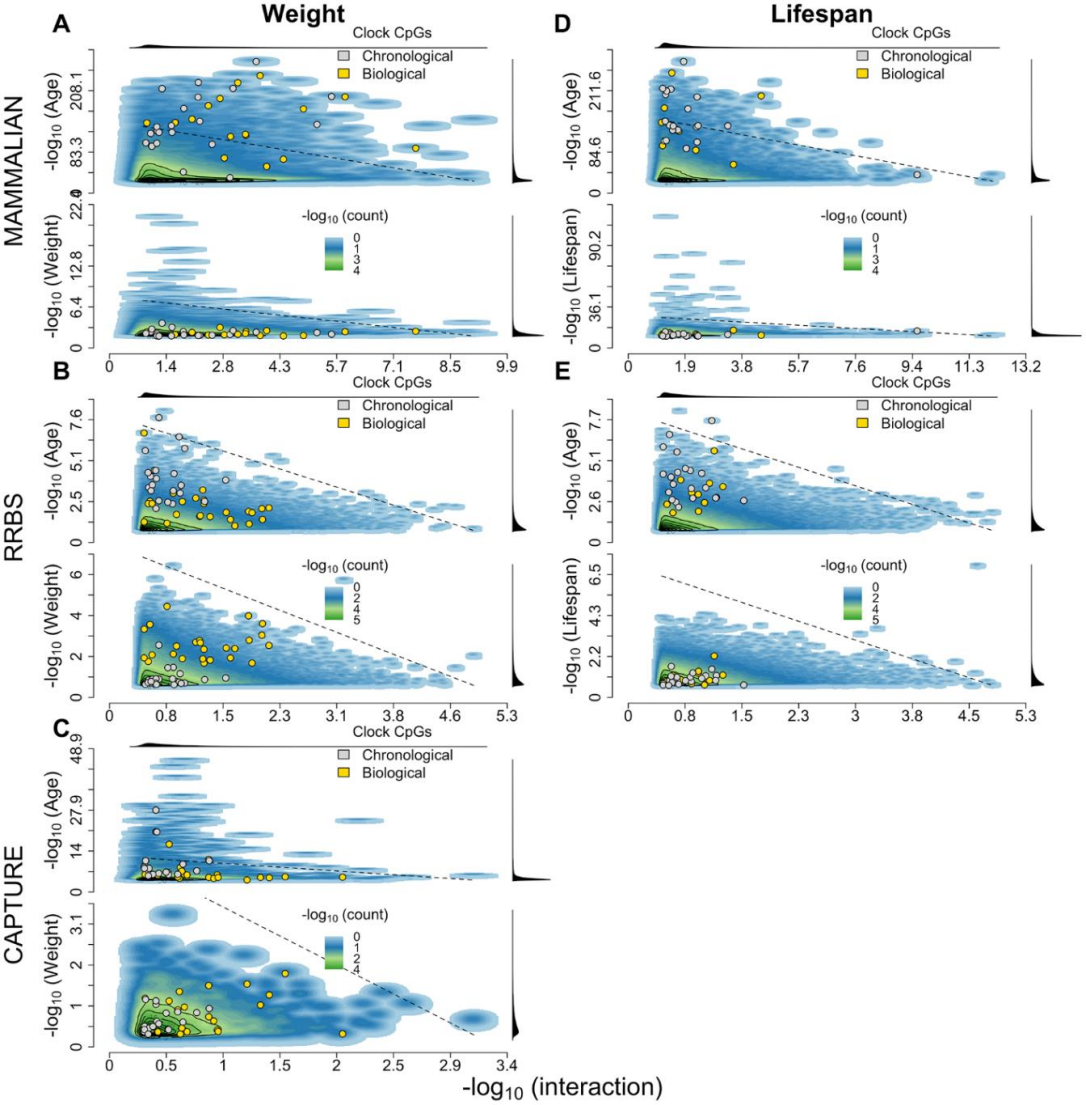
**2D density distribution of biological age EWAS p-values.** (**A**–**E**) The top panel corresponds to the mammalian methylation array, the center panel to the capture sequencing and the bottom panel to the RRBS dataset. The x-axis shows the likelihood improvement of the nested model containing age, the moderator variable and the age-moderator interaction against the model containing only chronological age, both with phylogenetically corrected errors. Panels (**A**–**C**) use weight as the moderator variable while (**D**–**E**) use lifespan. The top and bottom y-axes in each panel correspond to the p-values for age and for the moderator variable with phylogenetically corrected errors, respectively. The marginal densities of each p-value distribution are plotted in the top and right margins of each plot. The sites that contribute to the penalized regression chronological (gray) and biological age (yellow) clocks are annotated within each plot. The dotted lines correspond to linear combinations of -log p-values corrected for inflation (i.e., median of empirical p-value distribution divided by median of uniform distribution [~0.5]).

A site-by-site examination of biological age signals revealed few biological age candidate sites both before and after correcting for phylogeny (Figure 5). Because sites more tightly associated with chronological age produce smaller interaction effects, the p-values for age and the interaction term between age and the moderator variable are not independent. Nevertheless, we report a general depletion of sites that are simultaneously significant for age and the interaction term between age and the moderator, indicating that very few sites display true potential biological age effects. Even then, the sites that make up the biological age epigenetic clocks tend to be the best candidates for biological age effects (Figure 5), highlighting that the phylogenetic and ordinary penalized regression models are selecting for the correct features of their component sites. Only the RRBS biological age epigenetic clock displayed an excess of sites independently associated with weight (Figure 5B, second panel). Indeed, RRBS presents the largest inflation of weight-associated p-values of all datasets, especially given its small sample size (Figure 5), highlighting that it is potentially composed of more phylogenetically concordant sites than the two other datasets.

### Gene ontology enrichment in biological age loci

We performed gene ontology (GO) enrichment analysis on the set of genes 5kb upstream or downstream of the 50 top biological age candidate loci. As an insufficient number of significant sites were found to be associated with biological age, the top candidate loci were arbitrarily picked based on a linear combination of p- values of age and the interaction between age and the moderator, relaxing significance to include 50 sites (Figure 5). As expected, due to the small number of shared sites between datasets, the top ten loci with the smallest biological age p-values in each dataset did not colocalize (Supplementary Figure 4). No GO terms were found to be significant in the capture sequencing dataset. Both the RRBS and the mammalian methylation array datasets were significantly enriched for GO terms related to skeletal and nervous system morphogenesis such as “GO:0007389 pattern specification process”, “GO:0048598 embryonic organ morphogenesis” and “GO:0021675 nerve development”. Enrichment for the same or similar terms was reported in the respective studies for chronological age. Of note, the main loci driving these terms in both the RRBS and mammalian methylation array datasets are the *HOX* gene clusters (in particular *HOXA1-10*), which have previously been identified as major markers of biological age in mice [36]. Albeit consisting of different positions, the *HOXA1-10* region is also represented in the capture sequencing experiment, but it was not found to be significant for biological age effects.

## DISCUSSION

All dogs are members of the same species, *Canis lupus familiaris*, and were domesticated from gray wolves about 15,000-30,000 years ago [38]. Moreover, most breeds were developed in the last 200 years [20], many from a small number of founders [39], and thus share a similar genetic background [20, 40], which makes differences in breed-associated lifespan all the more remarkable. In this study we ask whether dogs experience changes in their epigenetic age at a rate predicted by breed longevity. Using publicly available dog methylation datasets, we compare three methylation platforms and tissues using unified and comprehensive methods to create biological age epigenetic clocks. Our two main innovations consist of training epigenetic clocks directly on biological age estimates and incorporating phylogeny to ensure orthogonality from phylogenetically concordant variation. The study of biological age in the dog can reveal novel candidate genes for anti-aging interventions, both in canines and in other species, and guide individualized, geriatric veterinary care.

Our analyses show that the penalized regression algorithms can create cross-validating biological age clocks in the dog mammalian methylation array and capture sequencing datasets at the cost of adding a sizeable number of methylation markers to the prediction formula (Supplementary Figure 2). We speculate that this is partly because both datasets consist of independent age and phylogenetically concordant methylation markers, and a combination of both kinds of markers can resemble the signature of biological age. While not overfit due to cross-validation, inspection of these parameter-rich models reveals little correlation of individual markers with biological age, which hampers the interpretability of the clocks (Supplementary Figure 3). This comes in stark contrast to chronological age epigenetic clocks, which require fewer age-correlated sites for effective prediction (Figure 4). To circumvent these problems, we attempt to create sparser, phylogenetically corrected biological age clocks. The biological age signal from these new clocks is attenuated, supporting the hypothesis that we cannot create simple biological age clocks that are unconfounded by population stratification. While this result is expected because biological age clocks are constructed using breed standard data, the extent to which weight and, specifically, lifespan affect methylation rate changes could have been largely independent of the global phylogeny. RRBS has the greatest potential to detect biological age effects, as it features a much greater number of sites and is less biased towards chronological age determination. However, the poor cross-validation condition of the RRBS weight biological age epigenetic clock (Supplementary Figure 1) suggests that RRBS using DNA isolated from blood will produce unreliable biological age clocks, which potentially combine chronological age sites with lifespan-associated sites, instead of recapitulating true biological age (Figures 4 and 5). Alternative epigenetic clock formulations such as the epigenetic pacemaker and the BayesAge clock are consistent with the penalized regression clocks but less effective at incorporating phylogenetic correction.

While also attenuated by phylogenetic correction, the capture sequencing dataset in saliva is able to produce relatively sparse biological age clocks. Because the methylation sites present in the capture sequencing dataset are largely shared by the blood RRBS dataset, the ability to build a sparse biological age epigenetic clock in the capture sequencing dataset could be due to differences in tissue, although platform precision, performance and sample size cannot be ruled out. This result could also be driven by the presence of mixed breed dogs whose real weight was used in place of the breed standard. Also, we speculate that this difference could be due to the fluctuating cell composition [41] found in saliva versus blood during aging, or to the presence of epithelial cells, which are absent in blood. Both explanations suggest that cell fraction deconvolution would improve resolution for detecting biological age effects, perhaps even in the blood datasets. Additionally, we note that saliva samples are more susceptible to minor contamination effects by exogenous DNA. Unfortunately, no normal single cell tissue atlas has yet been produced for dogs, and the application of the human cell atlas to the dog methylomes is infeasible due to the absence of shared sites (Supplementary Figures 7, 8 and Methods).

Our results support the hypothesis that epigenetic biological age clocks trained on biological age estimates are better powered to detect biological age effects than a two-stage penalized regression of chronological age, or the equivalent analysis of chronological age residue variance (Figure 4B, 4D, 4F). The most likely explanation is that all datasets are heavily enriched for chronological age markers and, therefore, a first step penalized regression using chronological age as a response alone will not ensure selection of the optimal combination of markers that maximize residual correlation with lifespan or weight. Additionally, biological age epigenetic clocks created using weight display more significant interaction terms than those created using lifespan, even though the two measurements are highly anticorrelated [15]. This effect could be driven by the greater variability of weight measurements across breeds, but it is also possible that the weight clocks are subjected to minor, additional effects unrelated to longevity.

Biological age estimates are only feasible if lifespan differences are consistent and predictable in the organism of interest, which is not the case for humans and most other mammalian species. Using this rationale, we hope to uncover methylation markers reflecting biological age that could be translated across species. However, we find no individual sites that display clear biological age signals that are common to all datasets. This suggests that biological age is not reflected in the portion of the blood or saliva methylome analyzed by current platforms, or that it only affects a modest number of sites in relation to pure chronological age. We posit that the lack of individual sites displaying strong biological age signals also results in the general inability to create sparse epigenetic biological age clocks. Of note, the saliva capture and the RRBS datasets have a reduced sample size and therefore could be underpowered to detect biological age effects compared to the mammalian methylation array. Whole methylome sequencing of additional tissues in the dog could offer new insights into the molecular basis of biological age and permit the development of new platforms, independent of phylogenetic effects, that are targeted at evaluating biological age. However, more invasive tissue sampling from healthy dogs of different ages and breeds would be required, which poses significant ethical issues.

The overall absence of biological age markers unconfounded by phylogeny across established platforms in a species with such breed-associated variable lifespans as the dog challenges some hypotheses regarding intra-species methylation accumulation in mammals. The phenomenon that the rate of accumulation of methylation adjusts to stages in the life of an organism, resulting in acceleration or deceleration if different organisms age at different speeds, could be much more difficult to detect than originally hypothesized in dogs and, potentially, other organisms as well. As such, the applicability of methylation markers to predict longevity in healthy individuals should be assessed on a species-by-species and tissue-by-tissue basis. Further studies specifically targeting biological age in dogs and other species with known and diverse times of death will be needed to quantify the effects of biological versus chronological age in the mammalian methylome.

## MATERIALS AND METHODS

### Data reprocessing and metadata regularization

We leveraged three previously published methylation studies in dogs that used different experimental designs: RRBS in dog blood (SRA: SRP065666), an application of the mammalian methylation array in dog blood (GEO: GSE223748 and https://mydata.clockfoundation.org/app/mammalian-consortium-data-browser), and capture sequencing in dog saliva (https://www.tandfonline.com/action/downloadSupplement?doi=10.1080%2F15592294.2022.2069385&file=kepi_a_2069385_sm0108.zip). SNP data for the capture sequencing experiment were requested from the authors. The three studies offered pre-processed and filtered beta values readily available for analysis. We chose, however, to re-process the RRBS dataset to recover more methylation sites and improve comparability. After adapter trimming [42], the raw reads were aligned against the CanFam3.1 Tasha reference [43] and beta values were calculated using the Bismark [44] suite, excluding the first and last three nucleotides, as they are part of the restriction site and reflect biased nucleotide compositions.

bismark -N 1 –output_dir ${output_dir} ${input_fastq} && bismark_methylation_extractor –ignore 3 –ignore_3prime 3 –bedGraph –output ${output} ${input_bam}.

Methylation sites missing in at most four samples were tabulated and resulted in a total of 244,334 sites. We found over 98% Pearson correlation between our methylation calls and the original study. SNPs were called from aligned reads using hard heterozygous allele balance (AB) thresholds of 0.1<AB<0.9 and filtered for minor allele frequency > 0.1 to a total of 158,989. All C **→**T and A **→**G substitutions were discarded.

In addition to the metadata provided by each study, we complemented the information tables with breed-based expected lifespan using, if possible, the information published in Horvath et al. [3] and complementing the missing breeds with AKC [45] records.

### Construction of phylogenetic variance-covariance and distance matrices

In the analyses where SNPs could be extracted from the corresponding platform (RRBS and capture sequencing), we used Gower distances between samples to create global phylogenies which were then converted into variance-covariance matrices using the vcv function in the R ape package. In the case of the mammalian methylation array, where no SNP panel was available, we inferred population stratification based on breed. First, the average distance between all samples belonging to all possible breed pairs within a reference breed topology was calculated. Then, the resulting tree was subsampled to contain the breeds represented in the mammalian methylation array and the distance value of each breed broadcasted to all samples belonging to that breed (Supplementary Figure 6). Three out of ninety-four breeds were not represented in the reference topology, so a proxy breed from the same putative clade was assigned in their stead (English Setter → Irish Setter, Jack Russel terrier → Glen of Imaal terrier, Weimaraner → Wirehaired Pointing Griffon). Pagel’s lambda was calculated on this canonical phylogeny using the phytools [46] R package. Mantel tests between methylation and genetic variance-covariance matrices were calculated with the mantel.test function provided in the R ape package using 100,000 permutations. To correct for degeneracy in the resulting matrix, we raised each symmetrical entry of the matrix to a random power very close to one of the form *F*~*N*(1, 0.01) so that ${D^{\prime }}_{ij}={D^{\prime }}_{ji}=D_{ij}^{F}$ where *N* is a normal distribution draw, *D* is the original matrix and *D*′ is the resulting jittered matrix. Distance matrices were converted to variance-covariance matrices using the vcv function in the R ape package. Note that this approach allows for an arbitrary shrinkage of the determinant of the jittered matrix, which will ultimately influence the magnitude of the regression likelihood. Finally, to ensure positive semi-definiteness, we applied the nearPD R base [47] function to the resulting matrix (Supplementary Figure 6).

### Phylogenetic penalized regression and epigenome-wide association study

Before applying penalized regression, we performed two corrections to the methylation matrix based on the two previous methods sections: first, we linearized the methylation matrix, site by site, using the Box-Cox transformation implemented in the R MASS [48] package. Second, we performed a decorrelation transformation by multiplying the linearized methylation matrix and response variable with the inverse Cholesky-factored variance-covariance matrix to account for phylogenetic dependence between samples. To validate this procedure, we proved that it is possible to use the glmnet proximal gradient descent framework with decorrelated, generalized errors (Supplementary Text 1 and Supplementary Figures 9, 10). We limited the number of markers that make up any clock to ≤ 25, as this value lies in the interval between the number of markers corresponding to the minimum cross validating penalty value and the penalty value one standard error above it (Supplementary Figure 1). All biological and chronological age epigenetic clocks, except for RRBS moderated by weight, passed ten-fold cross-validation both under ordinary and generalized least squares error models (Figure 4C, 4D and Supplementary Figure 1). Generalized-penalized regression models were then deployed using the R glmnet [49] package with fifteen-fold cross-validation, using age as the dependent variable. Given the diverse breed composition of the three datasets, ten-fold cross-validation separated all the components of more than one breed into a single cross-validation fold, ensuring non-redundancy between training and test dataset partitions. Our results were robust to permutation of the folds.

We applied independent, site-wise generalized least squares (GLS) regression models using methylation as the response variable and sex as a covariate. We implemented an exact solution to GLS regression to bypass the calculation of the inverse variance-covariance matrix for every site, which is standard in R packages such as gls. The interaction term was explicitly modeled to be a quotient in the case of lifespan moderation and a product for weight. Specifically, we tested whether the addition of an interaction term such as lifespan or weight, improves the relationship between methylation and chronological age. For any site i let M1, M2 and M3 be three separate linear models containing the following terms:


$$M_{1}:=BC(methylation_{i})=Age+Sex+\varepsilon_{i}$$

$$\begin{array}{l} M_{2}:=BC(methylation_{i})=Age+Moderator\\ \;+Moderator:Age+Sex+\xi_{i} \end{array}$$

$$M_{3}:=BC(methylation_{i})=Moderator+Sex+\delta_{i}$$

∀ i ∈ {1, 2, …, N}, N :number of CpG sites

$$\begin{array}{l} BC(X)\sim Box-Cox\;transformation\;of\;variable\;\\ \;X\;(methylation) \end{array}$$

$$\begin{array}{l} Residuals\;\varepsilon_{i},\;\xi_{i},\;\delta_{i}\sim N(0,\;\Sigma ),\\ \;0:vector\;of\;0s;\;\Sigma :phylogeny\;variance\\ \;-covariance\;matrixa \end{array}$$

The increase in likelihood between the model M2 and M1 as well as the p-value of the interaction term in M2 allowed us to query the linear regression model for biological age signals. Additionally, the interaction term in M2 was compared against the isolated lifespan term in model M3 to rule out an independent effect of lifespan in the regression.

### Epigenetic pacemaker and bayesAge

We re-implemented the epigenetic pacemaker model described by Snir et al. [33] in R including cross-validation. Additionally, we implemented a version of the BayesAge [50] inspired age determination algorithm described by Mboning et al. [32]. In brief, for every relevant site, a non-parametric curve relating methylation and age is built in a training cross-validation fold using locally estimated scatterplot smoothing (LOESS) [51]. These curves are queried for the probability of a test sample being any age given its methylation status. This algorithm can be interpreted as a realization of a leave-one-out cross-validation of the methylation-age LOESS regression fit and can be readily extended to *n*-fold cross-validation by doing bulk prediction in test fold *n* instead of a single-sample. The final age likelihood of a sample is calculated by multiplying the probabilities for any given age over all sites.

As we do not possess a breakdown of methylated and unmethylated reads, we modified the algorithm to handle M/beta values. Instead of using a (negative) binomial link function between age and methylation, we simply use the LOESS prediction and standard deviation values for a given methylation value to create a pseudo-probability distribution of age (Supplementary Figure 5). Particularly, we model *P*(Age = *x*)~*N*(*m*, *s*) where *m* is the predicted age given a methylation value and *s* is the standard deviation of the LOESS curve at point *m*. We note that, contrary to the binomial distribution, this approach accounts for the heteroskedasticity of the LOESS curve but does not account for the within-sample coverage variability as this information is unobtainable from M/beta values.

### Cell type deconvolution and gene ontology analysis

We first attempted to use the human blood methylation atlas to perform cell fraction deconvolution on the RRBS and mammalian methylation array datasets. We observed that standard deconvolution algorithms such as DeconRNASeq in R returned similar outputs if a proper methylation matrix or a matrix of random numbers were used as an input (Supplementary Figure 7). To assess the possibility to perform deconvolution using the shared sites between the mammalian methylation array and the human methylation atlas, we reframed the deconvolution as a constrained regression problem. Then, we derived multiple regression p-values which assess whether the complete regression model implies an improvement from an only-intercept model. We used the R quadprog library to estimate the regression coefficients enforcing the restrictions that all coefficients should be positive and sum to one. We observe that deconvolution regression models for blood are not significant in dogs and barely reach significance in humans when applied to the subset of CpGs present in the mammalian methylation array (Supplementary Figure 8). Sheep methylation data [4] and data from a blood capture sequencing methylation assay in Labrador retrievers [12] were added for confirmation.

We performed gene ontology (GO) enrichment analysis using the web server webGestalt [52] on the set of significant genes derived from each dataset. Each ontology search was performed against a background of all genes represented in the corresponding dataset. We used human gene ontology networks in place of the dog, as they are more densely populated with curated terms.

### Data and materials availability

All data needed to evaluate the conclusions in this paper are present in the paper, code and/or the Supplementary Materials. The raw data for the three datasets can also be obtained from: SRA: SRP065666, GEO: GSE223748, https://mydata.clockfoundation.org/app/mammalian-consortium-data-browser, https://www.tandfonline.com/action/downloadSupplement?doi=10.1080%2F15592294.2022.2069385&file=kepi_a_2069385_sm0108.zip. The code used to analyze the processed data is available at https://github.com/aserresarmero/bioage_methylation.

## Supplementary Material

Supplementary Figures

Supplementary Text
