# Supplementary material for: Co-analysis of methylation platforms for signatures of biological aging in the domestic dog reveals previously unexplored confounding factors
Source: Aging (Albany NY). 2024 Jul 9;16(13):10724–48. doi: 10.18632/aging.206012 (PMC11272130; doi:10.18632/aging.206012)
Supplement: Supplementary Figures [file aging-16-206012-s001.pdf]

SUPPLEMENTARY FIGURES

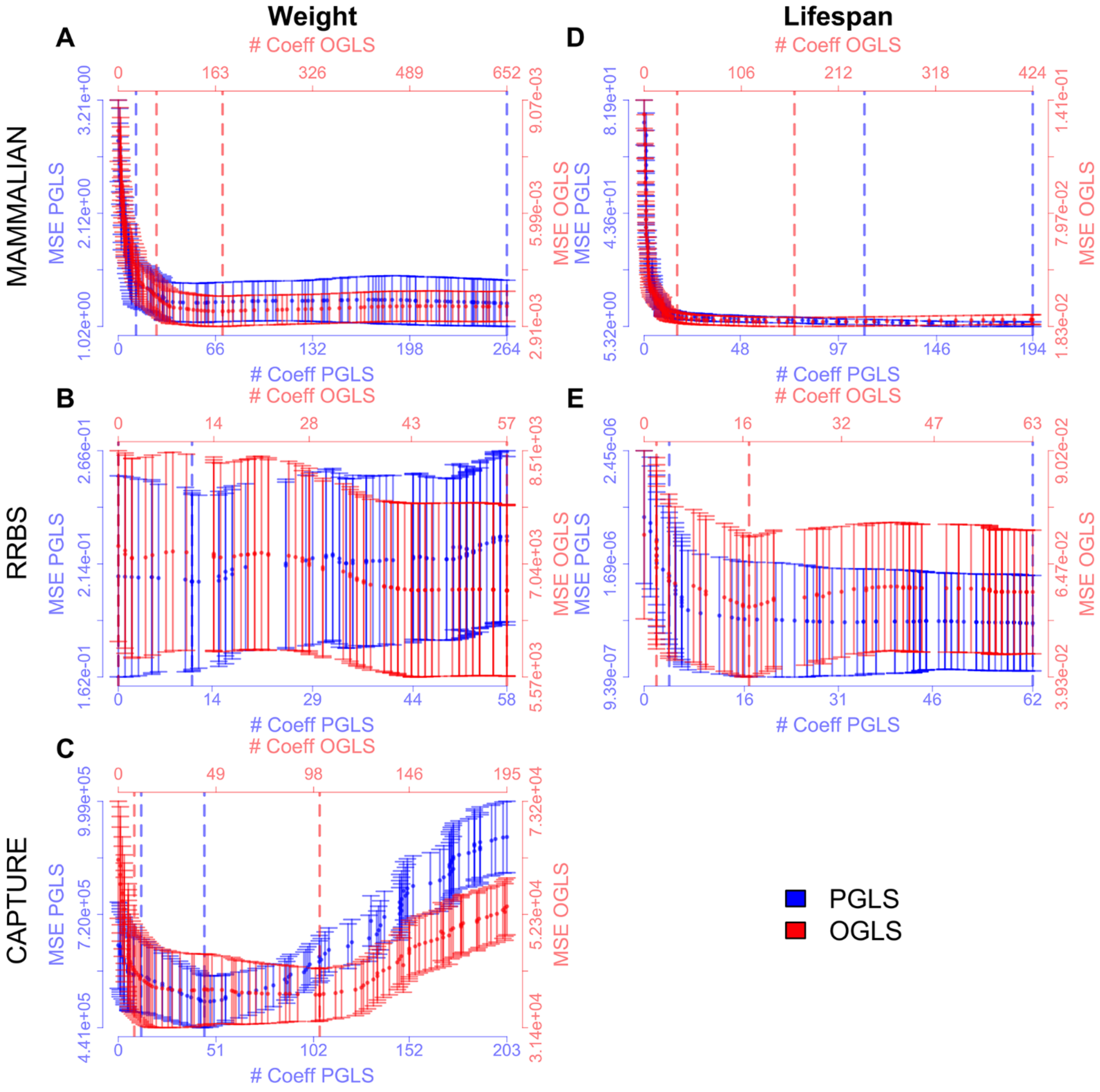

Supplementary Figure 1. Ten-fold cross validation plots for the phylogenetically corrected (blue) and ordinary least squares (red) biological age, weight, and lifespan epigenetic clocks. (**A**–**C**) correspond to weight clocks for the mammalian array, RRBS and capture sequencing datasets. (**D**, **E**) correspond to lifespan clocks for the mammalian array and RRBS datasets. The top and bottom horizontal axes in every plot correspond to the number of coefficients for each regression type while the left and right vertical axes correspond to the cross-validation mean squared error. The dashed vertical lines show the number of coefficients of the model associated with the minimum cross-validation error (optimal) and that of the model with a penalty one standard error above it (sparsest). Note that a threshold of 25 coefficients used in the manuscript generally falls within that interval. Importantly the RRBS weight clocks in (**C**) display a flat profile denoting that a zero coefficient, intercept-only model is close to equivalent to a heavily parametrized one and therefore the regression could be overfitted.

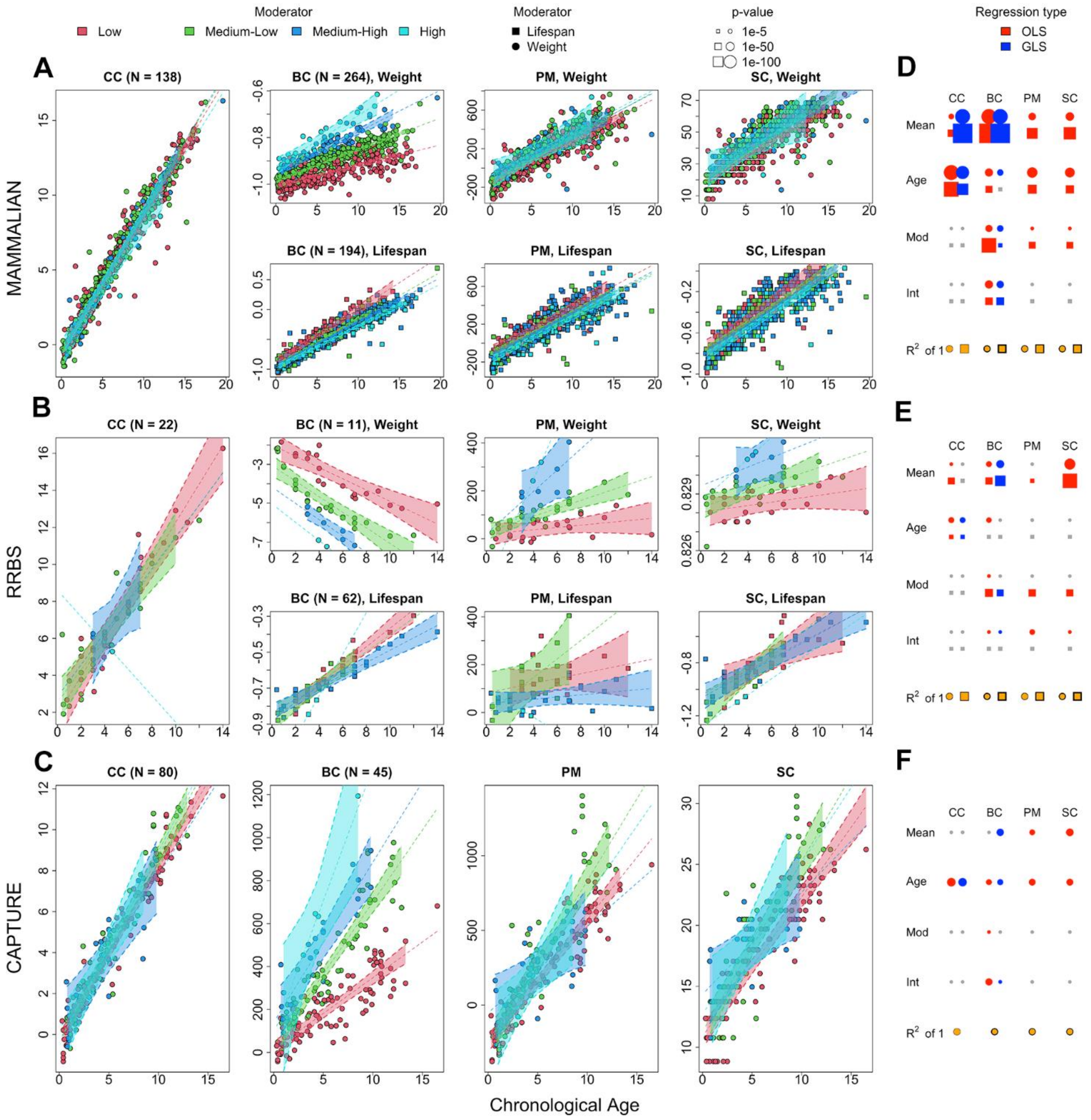

Supplementary Figure 2. Statistics of chronological and biological non-sparse (maximum number of cross-validating coefficients) epigenetic clocks. Rows from top to bottom correspond to the mammalian methylation array, RRBS, and capture datasets respectively. (**A**–**C**) The first, second, third and fourth panels in each row represent the different epigenetic clocks. CC: penalized generalized least squares regression trained on chronological age, BC: penalized generalized least squares regression trained on biological age, PM: epigenetic pacemaker trained on biological age data, SC: BayesAge algorithm trained on biological age data. The trend lines and 99% confidence intervals are derived from an ordinary least squares model. Any split panels depict the use of weight or lifespan as a moderator as described in the panel and legend. (**D**–**F**) The rightmost plots of each row depict the significance of each regressor in the corresponding dataset, with circle radii proportional to -log p-value (blue: phylogeny corrected least squares, red: ordinary least squares, gray: non-significant), the yellow-colored fraction of the area of the bottom squares depicts the regression R^2 values derived from the ordinary least squares model.

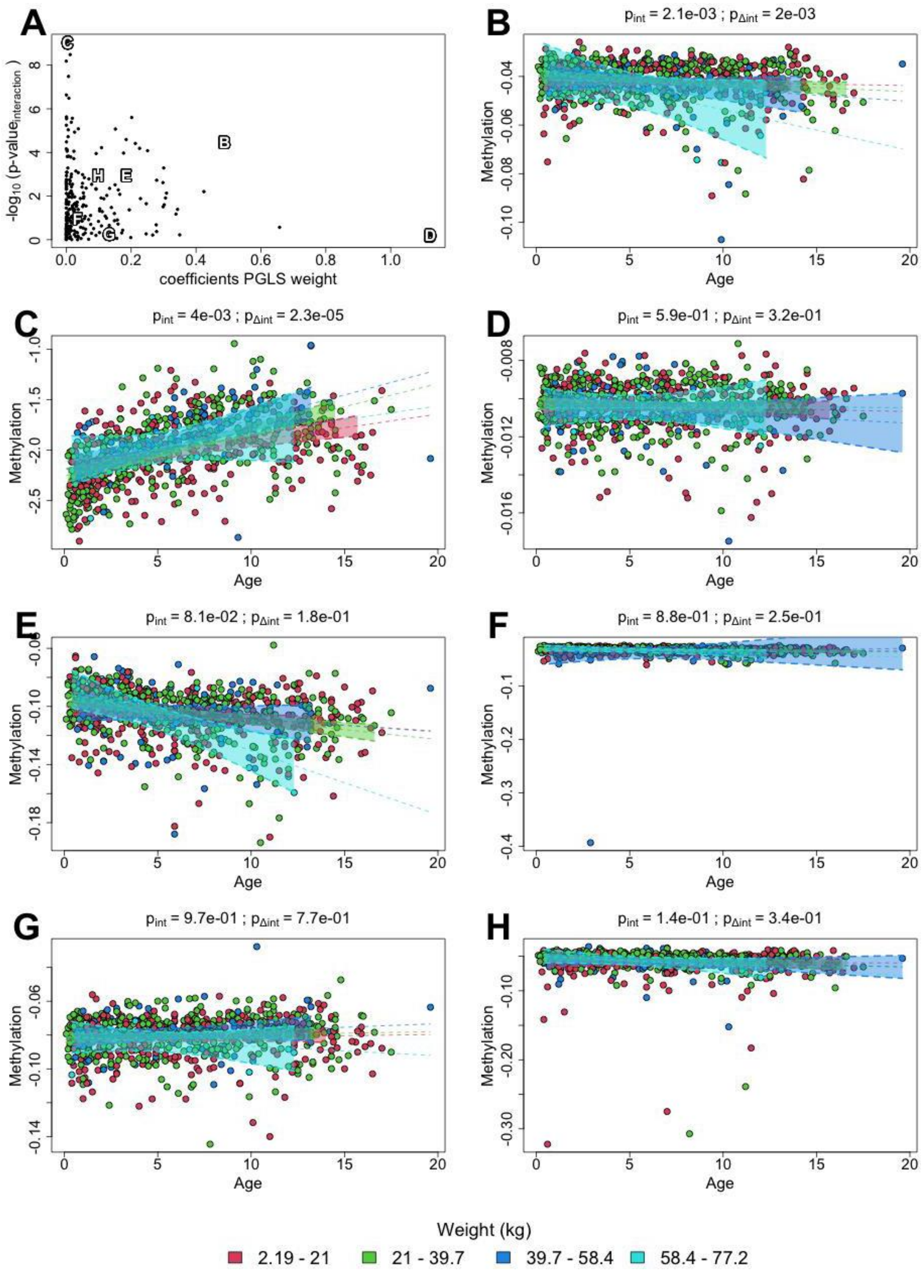

Supplementary Figure 3. Example of the methylation dynamics of specific CpG sites (N=264) that constitute the non-sparse, mammalian array, biological age clock constructed using weight. (**A**) Scatter plot of p-values (y-axis) for the interaction using the model described in Figure 5. The x-axis represents the coefficients assigned to those sites in a non-sparse, penalized regression model. A few key and randomly selected sites with active regression coefficients are marked with letters (**B**–**H**) and dissected in the following panels. (**B**–**H**) Scatter plots of methylation values (y-axis) versus age (x-axis) for the sites highlighted in A. Points are colored by equal length weight bins. The heading of each panel shows the p-value for the interaction term (p_{int}) and the p-value for the model likelihood increase after adding the interaction and weight main effects ($p_{\Delta int}$). As evidenced by the low interaction p-values of most sites contributing to the regression model and the examples shown, the non-sparse model may be much less robust and difficult to interpret than its sparse counterpart.

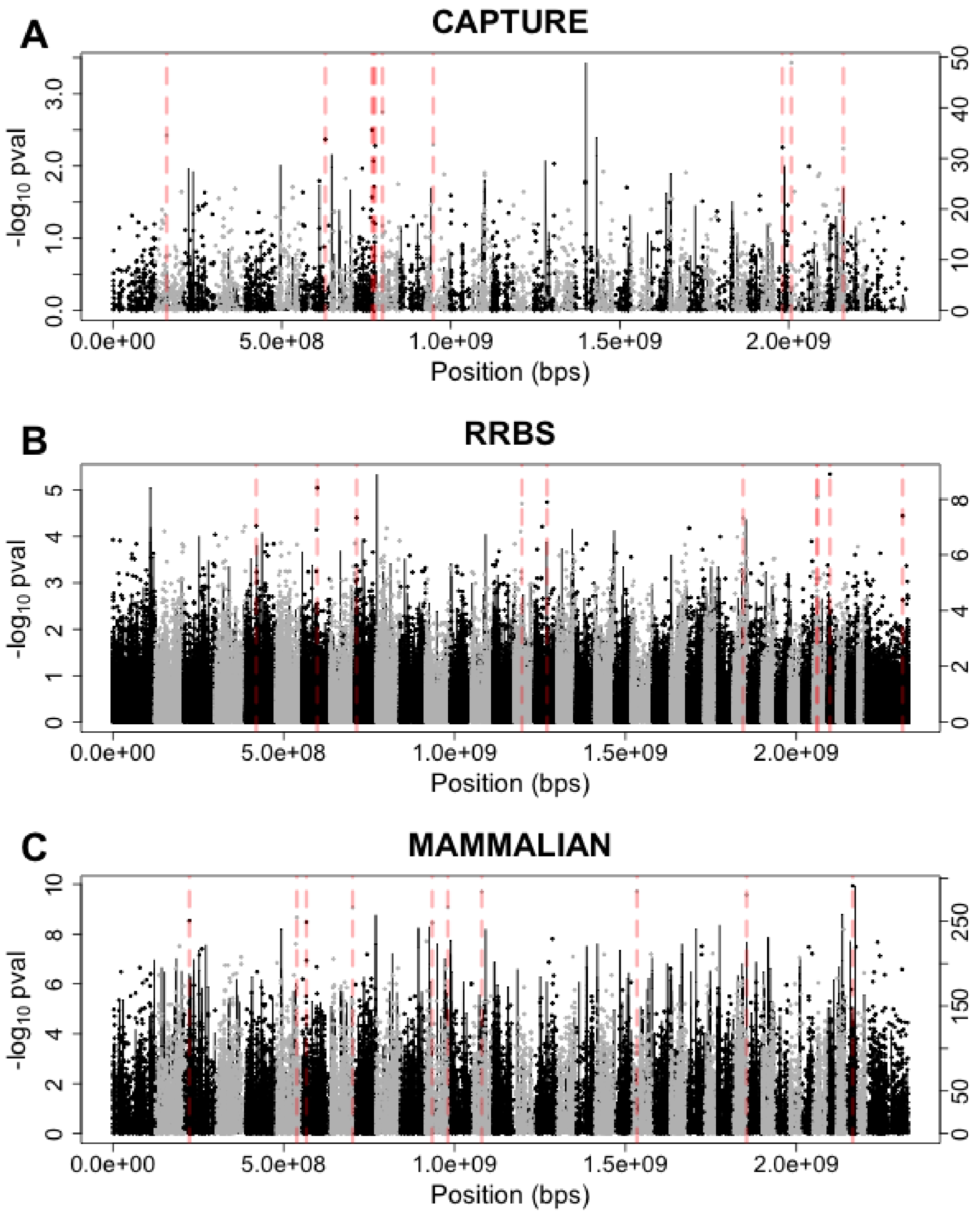

Supplementary Figure 4. Manhattan plot showing p-values for biological age signals using weight as a moderator. (**A**–**C**) The p-values for the interaction effects are represented by points corresponding to the right y-axis while the p-values for association with age are represented in the right y-axis. The x-axis shows the cumulative position along the genome, where each chromosome in ascending order is represented by alternating colors. The red, dashed lines mark the ten top interaction p-values in each dataset. No top 10 interaction locus in a dataset is within a Mb of a top 10 interaction locus of another dataset, meaning that the most suggestive biological age candidates in each dataset do not colocalize.

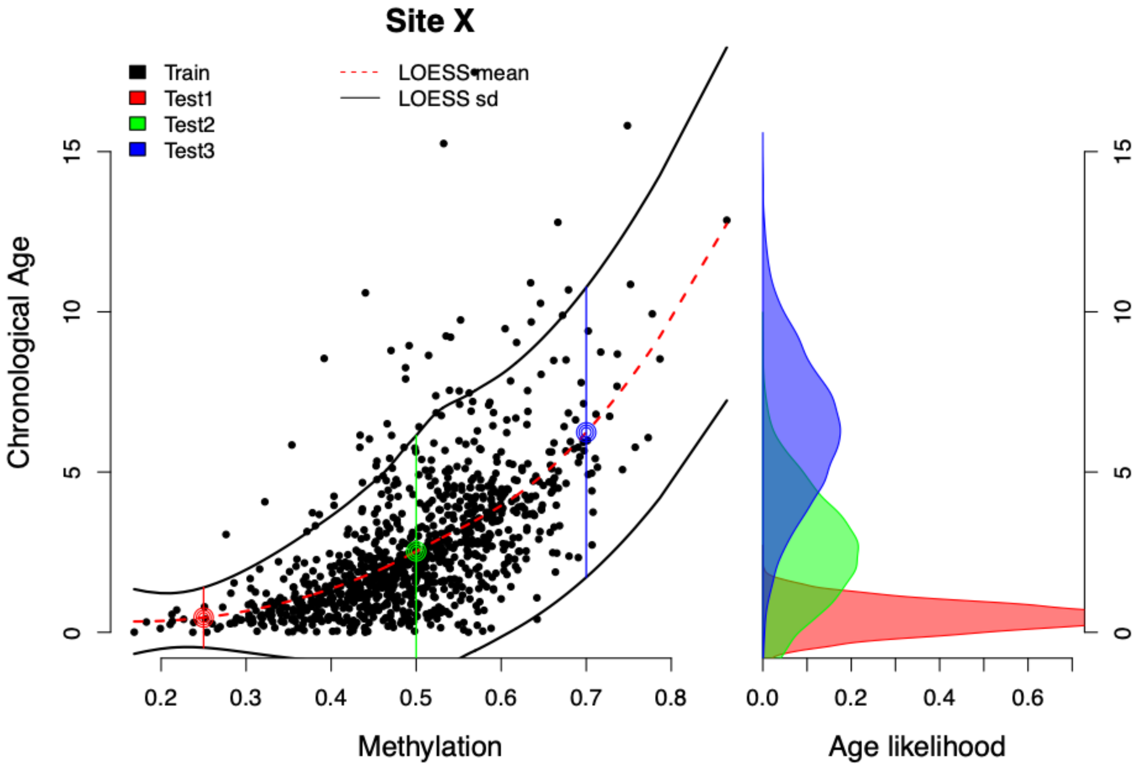

Supplementary Figure 5. Schematic representation of our adaptation of the scAge-inspired algorithm by Mboning et al., BayesAge. For site X, a non-parametric LOESS curve is fitted based on a training data fold (left panel). Means and standard deviations for a test fold are predicted from the LOESS curve (in this example test samples 1 [red], 2 [green] and 3 [blue]). Age likelihoods are then modeled after normal distributions for the predicted test fold means and standard deviations (right panel). The age likelihoods of every site are then multiplied together to extract the posterior sample age credible intervals and maximum likelihood values.

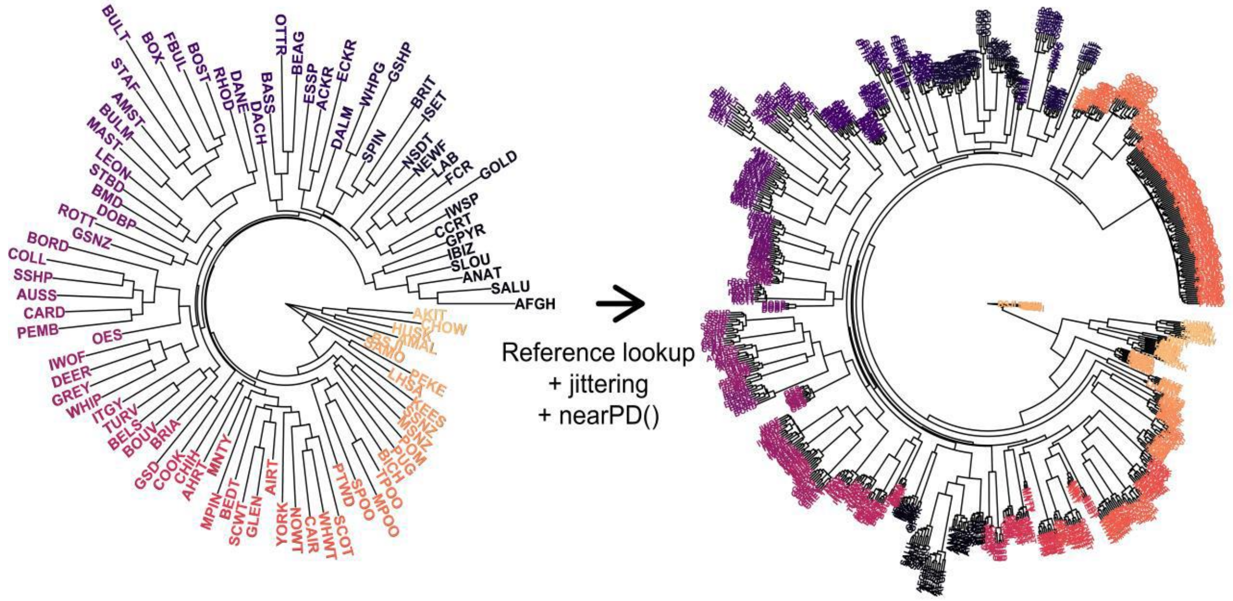

Supplementary Figure 6. Neighbor joining tree of the reference distance matrix constructed by averaging the distances derived from Parker et al. 2017 (left) and non-degenerate tree for the mammalian methylation array (right). Breeds are assigned colors to match labels between the two trees. The jittering and nearPD() processes are described in the Methods.

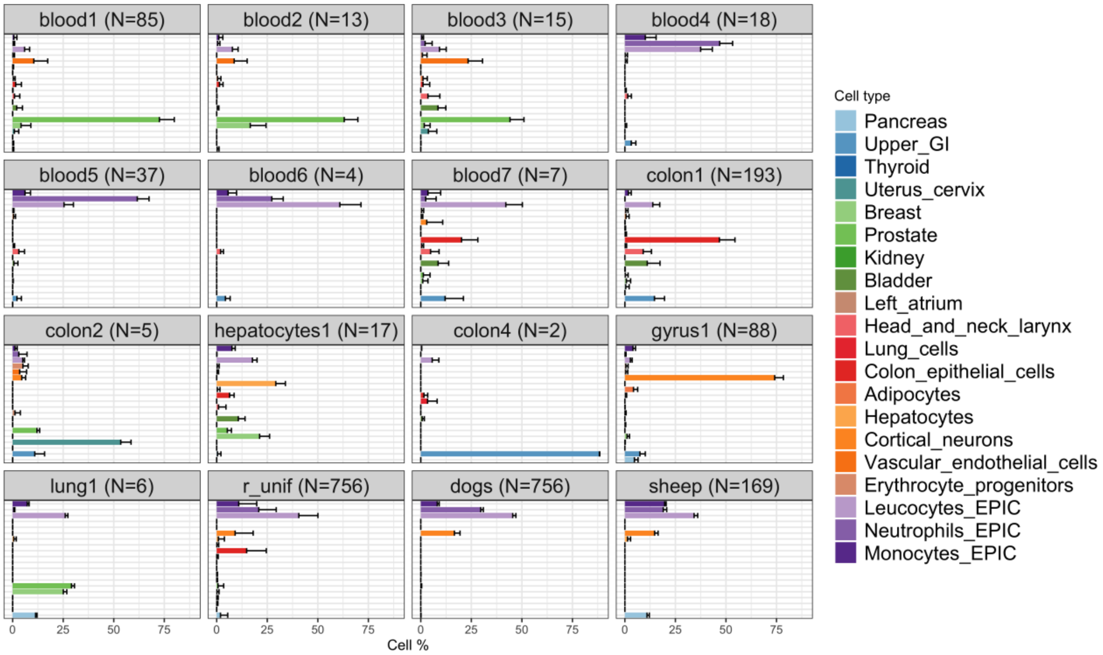

Supplementary Figure 7. Deconvolution results from 270 sites shared between the mammalian methylation array and the human methylation atlas. The numbers on top of each panel correspond to the number of samples per tissue/species. The three last panels correspond to a 756 x 270 matrix of random uniform numbers (r_unif), the dog methylation matrix and sheep methylation matrix, respectively, and are noticeably similar.

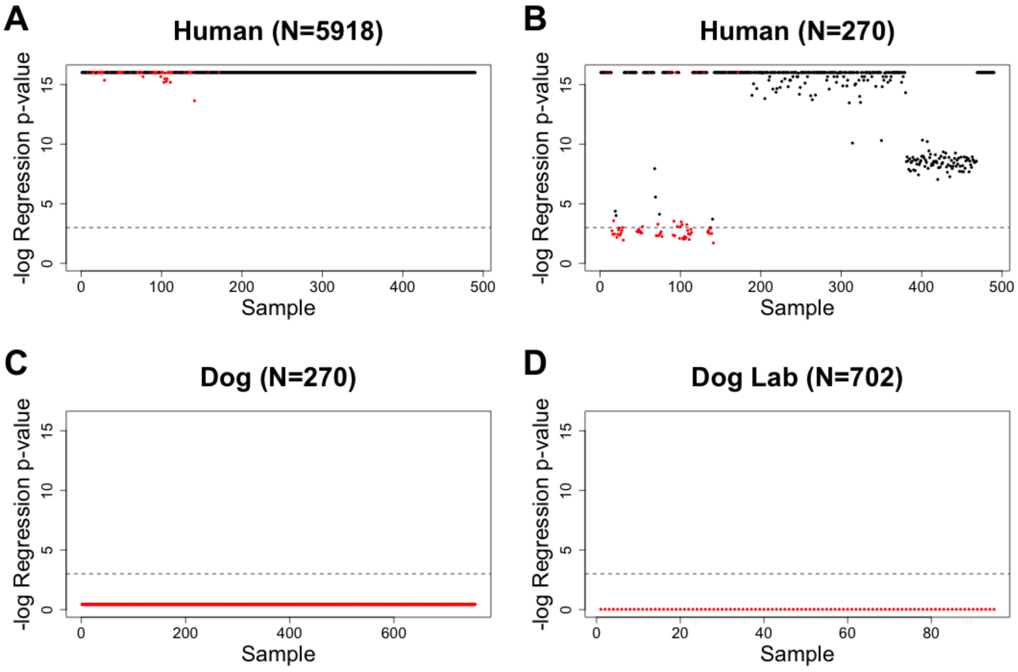

Supplementary Figure 8. Fisher-Snedecor multiple regression p-values with cell fractions as the dependent variable for each sample on the x-axis. All blood samples are colored in red but note that the human panels contain additional tissues in black. (**A**–**D**) The four panels correspond to the complete human methylation atlas, the intersection between human methylation atlas and mammalian methylation array in humans and dogs, and a blood capture sequencing methylation assay in Labrador retrievers, respectively. The numbers on top of each panel correspond to the number of methylation sites used for deconvolution and are always a subset of the complete human methylation atlas (N=5918).

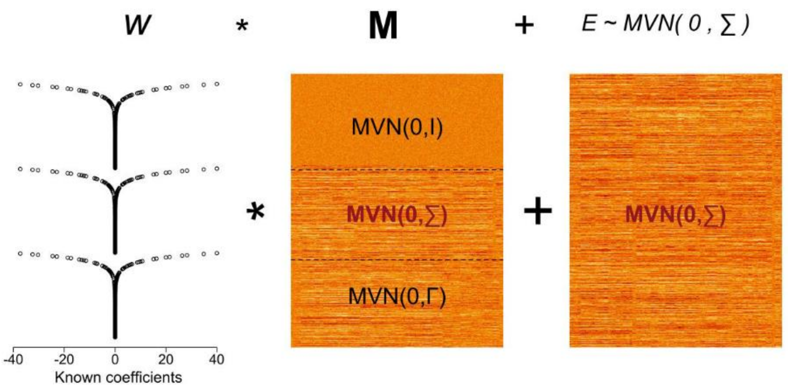

Supplementary Figure 9. Schematic representation of the regression model used to calculate the dependent variable *y* used for penalized generalized least squares simulation in Supplementary Text 1.

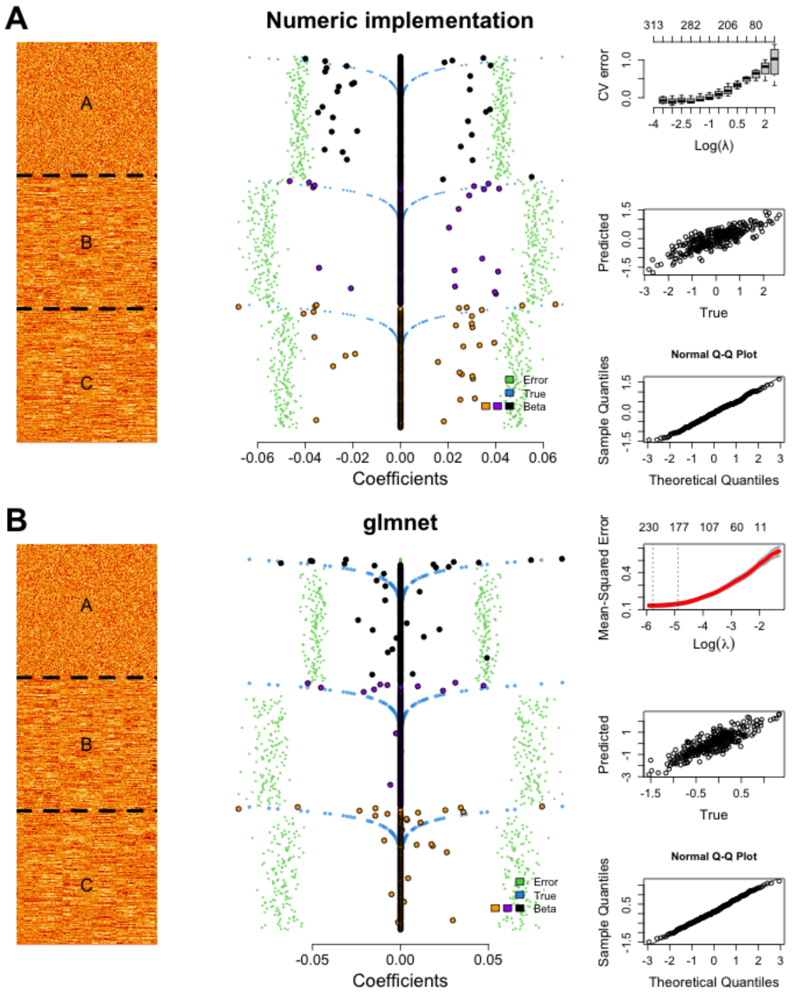

Supplementary Figure 10. Application of the numeric penalized GLS (top panel) and whitened glmnet regression (bottom panel) to the matrix *M* and vector *y*. (**A**, **B**) Right: model matrix aligned with center panel; Center: coefficients of the regression analysis. The solid points correspond to the non-zero predicted coefficients (black for matrix A, purple for matrix B and orange for matrix C) while the blue transparent points correspond to the real coefficients and the green transparent points to the error per coefficient. All coefficients have been projected to the same axis and are not to scale. Note that in both panels the model suppresses the coefficients in block matrix B and assigns them higher errors because they are distributed as the error matrix, highlighting the core idea behind penalized GLS.
