# Supplementary material for: Co-analysis of methylation platforms for signatures of biological aging in the domestic dog reveals previously unexplored confounding factors
Source: Aging (Albany NY). 2024 Jul 9;16(13):10724–48. doi: 10.18632/aging.206012 (PMC11272130; doi:10.18632/aging.206012)
Supplement: Supplementary Text [file aging-16-206012-s002.pdf]

SUPPLEMENTARY TEXT

Supplementary Text 1. Penalized-generalized gradient and simulations

We used the R glmnet package to perform penalized-generalized regression by first applying a decorrelating transformation on methylation and age, and subsequently using glmnet. While this approximation has been proven to be equivalent for numeric solutions to standard generalized least squares, to our knowledge there has never been an assessment of whether it extends to gradient descent solutions to penalized-generalized regression problems. Here we present proof that the penalized regression gradient matches the decorrelated generalized-penalized gradient. We support this conclusion with simulation work using both a numerical implementation of penalized, generalized least squares regression of complexity ~O(s^2n) and decorrelated glmnet ~O(sn) (where s is the number of coefficients [i.e., variables] to estimate and n the number of replicates [e.g., samples]).

Following the proximal gradient descent procedure to add and subtract an intermediate step variable β_kX, we attempt to find β which minimizes the error norm of a data matrix X and a vector y induced by the decorrelating matrix Ω^{-1}, with a constraint function $r(\beta)$ (e.g. ridge, lasso…): Where y, X, Ω^{-1}, β are the dependent variable, the independent data matrix, the inverse variance-covariance matrix and regression coefficients respectively:

$$\underset{\beta}{argmin} \| y-\beta \mathrm{X} \|_{\Omega^{-1}} + \lambda r(\beta) \Rightarrow \quad (1)$$

$$\begin{aligned} & \| (y-\beta_k \mathrm{X}) + (\beta_k \mathrm{X} - \beta \mathrm{X})_{\Omega^{-1}} + \lambda r(\beta) \Rightarrow \\ & \| (y-\beta_k X) \|_{\Omega^{-1}} + (\beta_k X - \beta X)_{\Omega^{-1}} \\ & \quad + 2(y-\beta_k X)\Omega^{-1} X(\beta_k - \beta) + \lambda r(\beta) \Rightarrow \\ & (y-\beta_k X)^T \Omega^{-1} (y-\beta_k X) + \| X(\beta_k - \beta) \|_{\Omega^{-1}} \\ & \quad + 2(y-\beta_k X)\Omega^{-1} X(\beta_k - \beta) + \lambda r(\beta) \end{aligned} \quad (2)$$

For shortness of notation, we define:

C_k = (y − β_kX)^T Ω^{-1}(y − β_kX) (scalar, independent of β) and $v_k^T = (y-\beta_k X)\Omega^{-1} X$ (vector, independent of β).

If the data matrix is distributed as a multivariate normal on its rows or columns, but not both simultaneously:

$$\begin{aligned} & \| \mathrm{X}(\beta_k - \beta) \|_{\Omega^{-1}} \Rightarrow \\ & (\mathrm{X}(\beta_k - \beta))^T \Omega^{-1} (\mathrm{X}(\beta_k - \beta)) \\ & < \| X \|_{\Omega^{-1}}^{op} (\beta_k - \beta)^T (\beta_k - \beta) \\ & \sim \frac{1}{\tau} (\beta_k - \beta)^T (\beta_k - \beta) \end{aligned}$$

where $\frac{1}{\tau} \geq \frac{1}{\| \mathrm{X} \|_{\Omega^{-1}}^{op}}$, τ is the gradient step and $\| \mathrm{X} \|_{\Omega^{-1}}^{op}$ is the operator norm of X under basis Ω^{-1}.

Note that none of the variable substitutions above depend on β. Substituting those back to (2) in terms of the unknown β and multiplying by τ to clear denominators:

$$\tau C_k + (\beta_k - \beta)^T (\beta_k - \beta) + 2\tau v_k^T (\beta_k - \beta) + \tau \lambda r(\beta) \quad (3)$$

To complete the square in (3) we subtract $-\tau^2 v_k^T v_k$, even though it will not contribute to the gradient:

$$\begin{aligned} & \tau C_k - \tau^2 v_k^T v_k + ((\tau v_k + \beta_k) - \beta))^T \\ & ((\tau v_k + \beta_k) - \beta)) + \tau \lambda r(\beta) \end{aligned} \quad (4)$$

Defining $w_k = \tau v_k + \beta_k$ and $D_k = \tau C_k - \tau^2 v_k^T v_k$ (scalar independent of β):

$$\underset{\beta}{argmin} D_k + (w_k - \beta)_2 + \tau \lambda r(\beta) \quad (5)$$

where (5) is compatible with regular penalized gradient descent. After applying the gradient and unpacking variables v_k and w_k, and provided that $r(\beta)$ is convex and separable (e.g., ridge and potentially lasso), the β_k update step becomes:

$$\begin{aligned} & \tau v_k + \beta_k \Rightarrow \beta_k + \tau(y - \beta_k \mathrm{X})\Omega^{-1}\mathrm{X} \\ & \Rightarrow \beta_k - \tau(\mathrm{X}\Omega^{-1})^T (\mathrm{X}\beta_k - y) \Rightarrow \\ & \beta_k - \tau(X^T \Omega^{-1T} X \beta_k - X^T \Omega^{-1T} y) \end{aligned} \quad (6)$$

Note that Ω is symmetric, so $\Omega^{-1T} = \Omega^{-1} = QQ^T$ (i.e. the Cholesky decomposition of Ω^{-1}), then replace $X^* = Q^T X$ and $y^* = Q^T y$ on (6) to achieve:

$$\beta_k - \tau(X^{*T} X^* \beta_k - X^{*T} y^*) \quad (7)$$

which is equivalent to the non-generalized gradient update formula. To support this lemma, we derive numerical solutions to penalized GLS regression and compare the results to glmnet's gradient descent (Supplementary Figures 9, 10). To this end, we construct a block matrix M, a set of weights *w* and an error matrix E where:

$$M = \begin{bmatrix} A \\ B \\ C \end{bmatrix}, A \sim N_{300}(0, I_{200x300}),$$

$$B \sim N_{300}(0, \Sigma_{200x300}),$$

$$C \sim N_{300}(0, \Gamma_{200x300}),$$

where $\mathbf{N}$ represents a draw from a multivariate normal distribution, I is the identity matrix and Σ and Γ are randomly generated variance-covariance matrices using the R ape package function rtree.

$w = [a \quad b \quad c]$, where *a*, *b*, *c* are identical weight vectors of length 200 drawn from $f(x)=s10^x$ at equally spaced points $x \subseteq [-5, 1]$ in 1/200 intervals and with randomized signs $s \sim B_{[-1,1]}(n = 200, p = 0.5)$, where $B_{[-1,1]}$ represents a draw from a binomial distribution with values −1 and 1.

$E \sim \mathbf{N}_{300}(0, \Sigma_{600x300})$, note that the error matrix E is distributed as the B block in M. Here, the entries in block matrix B are meant to exemplify measurements with high "phylogenetic signal", which need to be suppressed from the regression coefficient estimation even if they contributed to response variable *y* (Supplementary Figure 9).

We did not attempt to replicate exact results between the numeric penalized GLS and decorrelated glmnet, as that would entail a dissection of the glmnet package code. However, we detected the expected suppression of the decorrelated glmnet coefficients (Supplementary Figure 10). As such, we conclude that decorrelated glmnet regression can be used in place of the numeric solution to penalized generalized least squares.
